# Human Herpesvirus 6A Partially Suppresses Functional Properties of DC without Viral Replication

**DOI:** 10.1371/journal.pone.0058122

**Published:** 2013-03-05

**Authors:** Rasmus K. L. Gustafsson, Elin E. Engdahl, Oscar Hammarfjord, Sanjaya B. Adikari, Magda Lourda, Jonas Klingström, Mattias Svensson, Anna Fogdell-Hahn

**Affiliations:** 1 Department of Clinical Neuroscience, The Multiple Sclerosis Research Group, Karolinska Institutet, Stockholm, Sweden; 2 Center for Infectious Medicine, Karolinska Institutet, Stockholm, Sweden; 3 Faculty of Medicine, University of Peradeniya, Peradeniya, Sri Lanka; 4 Childhood Cancer Research Unit, Department of Women’s and Children’s Health, Karolinska Institutet, Karolinska University Hospital, Stockholm, Sweden; 5 Department of Preparedness, Swedish Institute for Communicable Disease Control, Solna, Sweden; Oklahoma Medical Research Foundation, United States of America

## Abstract

Human herpesvirus 6A (HHV-6A) is a common virus with a worldwide distribution that has been associated with multiple sclerosis. Whether HHV-6A can replicate in dendritic cells (DC) and how the infection might modulate the functional properties of the cell are currently not well known and need further investigations. Here, we show that a non-productive infection of HHV-6A in DC leads to the up-regulation of HLA-ABC, via autocrine IFN-α signaling, as well as the up-regulation of HLA-DR and CD86. However, HHV-6A exposure reduces IL-8 secretion by DC and their capacity to stimulate allogenic T cell proliferation. The ability to suppress DC functions important for activation of innate and adaptive immune responses might be one successful strategy by which HHV-6A avoids the induction of appropriate host defense mechanisms, and thus facilitating persistent infection.

## Introduction

Human herpesvirus 6 (HHV-6) was first isolated in 1986 from patients with lymphoproliferative disorders [1] and belongs to the β-herpesvirus subfamily of *Herpesviridae* that can establish lifelong latent infections in the host. HHV-6 isolates are classified into two distinct virus species, HHV-6A and 6B [2]. At the age of one year, most individuals have been exposed to either HHV-6A or HHV-6B [3], [4]. Whereas HHV-6B is the causative agent of exanthema subitum in young children [5], no disease has been clearly linked to HHV-6A. However, an association between HHV-6A infection and multiple sclerosis (MS) has been suggested [6]–[8]. Induction of adaptive autoimmune responses such as activation of autoreactive T and B lymphocytes against myelin proteins is believed to be one important mechanism for the demyelination seen in the central nervous system of MS patients [9]. Therefore it is important to investigate how immune cells handle HHV-6A infections.

One cellular receptor for HHV-6A is CD46 [10], a complement inhibitor molecule that is expressed on all nucleated cells [11]. Therefore, although HHV-6A/6B predominantly infects CD4+ T cells [12], [13], HHV-6A may have tropism for many different cell types, including immune cells like dendritic cells (DC). DC are a heterogeneous family of cells with specialized antigen presenting capacities [14] that have regulatory roles in both innate and adaptive immunity. DC respond to microbial and inflammatory stimuli, which initiates a process of cellular activation termed maturation. The process of maturation is associated with increased surface levels of human leukocyte antigens (HLA) and co-stimulatory molecules, and enhanced production of soluble inflammatory mediators such as type I interferon (IFN), interleukin (IL)-8, IL-6, tumor necrosis factor (TNF) and IL-12 [15], [16]. Previous studies report contradictory results on the capacity of HHV-6A and HHV-6B to replicate within DC and on the effect of HHV-6 on DC maturation and capacity to stimulate T cell proliferation [17]–[22]. Therefore, more detailed investigations are needed. In this study we addressed these issues and here report that DC inoculated with HHV-6A do not support productive viral replication. However, immature DC get partially activated upon HHV-6A inoculation, as evident by the up-regulation of CD86 and HLA-DR, and the IFN-α dependent up-regulation of HLA-ABC. Inoculation of DC with HHV-6A also modulated the secretion of cytokines in DC, as evident by augmented TNF and IL-12p70 secretion and reduced secretion of IL-8. Interestingly however, DC inoculated with HHV-6A were impaired in their capacity to stimulate allogenic T cell proliferation. This data provides novel insights in the process of immune modulation by HHV-6A.

## Materials and Methods

### Isolation of Peripheral Blood Monocytes and Generation of DC

Monocytes were isolated from buffy coats purchased from the Blood Transfusion Clinic at the Karolinska University Hospital. Briefly, monocytes were retrieved from the buffy coats using commercial kits (RosetteSep Human Monocyte Enrichment Cocktail solution, Stemcell, Grenoble, France; or Monocyte Isolation Kit II, Miltenyi Biotech GmbH, Bergisch Gladbach, Germany) and density gradient centrifugation on lymphoprep (Fresenius Kabi Norge AS, Oslo, Norway). For the generation of DC, purified monocytes were cultured in GlutaMAX containing RPMI 1640 medium (Invitrogen Gibco, Paisly, UK) supplemented with 10% fetal bovine serum (HyClone, Logan, UT, USA), 100 U/ml penicillin and 100 µg/ml streptomycin (Invitrogen Gibco), 6.5 ng/ml of recombinant human IL-4 (R&D systems, Minneapolis, MN, USA), and 250 ng/ml of recombinant human granulocyte-macrophage colony-stimulating factor (GM-CSF) (R&D systems or PeproTech, London, UK). RPMI 1640 as described above, but without IL-4 and GM-CSF is referred to as complete RPMI, whereas RPMI 1640 with no supplementary agents is referred to as incomplete RPMI. On day 3 of culture, half of the medium was replaced with fresh complete RPMI supplemented with IL-4 and GM-CSF. On day 6 or 7 of culture the cells were harvested and washed with incomplete RPMI medium. The cell population used for infections typically contained >70% CD14− CD1a+ live cells, as evident by flow cytometry (data not shown).

### HHV-6A Propagation and Infection

HHV-6A strain GS [1] was propagated in the T-cell line HSB-2. When the cytopathic effect (CPE) was >50% the cell culture was centrifuged for 10 min at 300 g and the supernatant was harvested and stored in aliquots at −80°C. The 50% tissue culture infective dose (TCID_50_) was determined by ocular inspection for cytopathic effect as previously described [23]. The TCID_50_ was calculated according to the method of Reed and Muench [24]. DC were inoculated with mock (supernatant from uninfected HSB-2 cells) or HHV-6A at multiplicity of infections (MOI) of 10^−1^–10^−4^ for 3h before they were washed with incomplete RPMI medium and further cultured in complete RPMI supplemented with IL-4 and GM-CSF in the presence of 5% CO_2_ and at 37°C. HSB-2 cells were infected in parallel as positive controls for infection. As positive controls for maturation, DC were cultured with 100 ng/ml lipopolysaccharide (LPS) (InvivoGen, San Diego, CA, USA) together with 500 U/ml IFN-γ (PeproTech). To assess the effects of IFN-α, uninfected DC were stimulated with IFN-α (100–500 pg/ml; PegIntron, SP Labo N.V., Belgium), and HHV-6A infected DC were cultured in the presence or absence of a neutralizing rabbit polyclonal antibody to human IFN-α (10 µg/ml; PBL interferon source, NJ) or normal rabbit serum (Santa Cruz Biotechnology Inc., CA, USA) as isotype control. DC were also incubated with replication incompetent HHV-6A supernatant that had been inactivated by UV irradiation for 20 min. This treatment eliminates the replication capacity to zero as evident by TCID_50_ assays performed as described above (data not shown).

### Assessment of HHV-6A Replication in DC

At 3 and 6 or 7 dpi, DC were analyzed by immunofluorescence assay (IFA) by fixation onto glass slides with a 1∶1 mixture of acetone and methanol at −20°C for 10 minutes, for blocking 5% goat serum and 3% bovine serum albumin in PBS was used, and the cells were stained with a primary mouse monoclonal antibody (MAb) specific to the HHV-6 glycoprotein gp116/54/64 (Advanced Biotechnologies, Columbia, MD, USA). The staining was visualized by an Alexa 633 conjugated F(ab’)2 fragment of goat anti-mouse IgG (Invitrogen). Staining with 4′,6-diamidino-2-phenylindole (DAPI) (Vector laboratories, Burlingame, CA, USA) was used to visualize the cell nuclei. Cover slips were mounted with mounting media (DAKO A/S, Glostrup, Denmark), and the slides were analyzed using a confocal microscope (Leica Microsystems, Wetzlar, Germany). Viral replication was in addition followed at 3 hours post infection (hpi) and at 1, 3, 6, and 14 days post infection (dpi) in cells and supernatants by real time quantitative polymerase chain reaction (Q-PCR) (7500 Fast Real-Time PCR System, Applied Biosystems, Warrington, UK) of the immediate-early gene as previously described [25]. DNA was extracted from cells or supernatants using a commercial bead based kit according to the manufacturer’s protocol (MagMAX-96 Viral RNA Isolation Kit, Applied Biosystems).

### Flow Cytometry

For flow cytometry, cells were labeled with the following MAbs: fluorescein isothiocyanate (FITC)-conjugated anti-CD83 MAb (eBioscience, San Diego, CA, USA), phycoerythrin (PE)-conjugated anti-HLA-ABC MAb (DAKO A/S), PE and Texas red (ECD)-conjugated anti-CD14 MAb (Beckman Coulter, Brea, CA, USA), peridinin chlorophyll-a protein (PerCp)-conjugated anti-HLA-DR MAb (BD Biosciences, Franklin Lakes, NJ, USA), PE and cyanin dye 7 (PE-Cy7) conjugated-anti-CD40 MAb, (BD Biosciences), pacific blue (PB)-conjugated anti-CD86 MAb (Biolegend, San Diego, CA, USA) and allophycocyanin (APC)-conjugated anti-CD1a MAb (BD Biosciences). To visualize and exclude dead cells from analysis, the cells were also incubated with an APC-Cy7-conjugated live/dead marker (BD Biosciences). All labellings were performed on ice for 30 min in PBS containing 2% FCS, 5 mM EDTA and 0.01% sodium azide (NaN_3_). All flow cytometric analyses were performed in a CyAn flowcytometer (DAKO Cytomation, Glostrup, Denmark) and data was analysed using the software FlowJo (Tree Star Inc., Ashland, OR).

### Cytokine Measurements

Myxovirus resistance protein A (*MxA)* gene expression is only induced by type I and III IFNs, and hence represent a sensitive indicator of total levels of bioactive type I and III IFNs in samples [26], [27]. For analyses of the total level of bioactive type I and III IFNs in the DC supernatant samples, we therefore used an *MxA* gene expression assay [28]. Briefly, levels of *MxA* mRNA were measured in cells of the lung cancer cell line A549, exposed to 100 µl supernatants collected from DC cultures at 3 dpi. DC had been exposed to HHV-6A, UV inactivated HHV-6A, LPS together with IFN-γ or mock. As standard we used IFNβ (Avonex®) (BiogenIdec Sweden AB, Stockholm, Sweden). The A549 cells were exposed to the samples for 6.5 h followed by lysis in lysis buffer containing 50% Nucleic Acid Purification Lysis Solution (Applied Biosystems) in PBS (Ambion, Stockholm, Sweden). The culture plate with cell lysate was stored in −80°C over night before mRNA was extracted (6100 Nucleic Acid PrepStation, Applied Biosystems). Thereafter, the mRNA was converted to cDNA using the High Capacity cDNA Transcription kit (Applied Biosystems) according to manufacturer’s instructions, prior to Q-PCR analysis (7500 Fast Real-Time System, Applied Biosystems) with primers and probes targeting the *MxA* gene and *18S* gene, where *18S* was used as endogenous control of mRNA expression. To further determine the specific types of type I and III IFNs produced by DC IFN-α, -β and -λ specific enzyme linked immunosorbent assays (ELISAs) were run on DC supernatants collected at 3 dpi using commercial kits (from Mabtech, Sweden for IFN-α and from R&D Systems, UK for IFN-β and IFN-λ).

Cytometric bead arrays (CBA) (BD Biosciences) targeting IL-8, IL-1β, IL-6, IL-10, TNF and IL-12p70 were run on DC supernatants collected at 6, 12, 24, 48 and 72 hpi. All cytometric analysis was performed with a Gallios flowcytometer (Beckman Coulter, Brea, CA, USA) and data were analyzed using the software FCAP (Soft Flow Inc., St. Louise Park, MN, USA).

### Allostimulatory Capacity of DC

The allostimulatory capacity of DC inoculated with HHV-6A or UV inactivated HHV-6A at MOIs of 10^−2^–10^−4^, LPS together with IFN-γ, or mock was evaluated by mixed lymphocyte reaction (MLR) with 3–30*10^2^ live DC and 10^5^ allogenic live T cells per well. Determination of cell numbers was performed using a phase contrast microscope (Nikon) after Trypan blue (Gibco) staining. CD3+ CD8+ and CD3+ CD4+ T cells were isolated from peripheral blood mononuclear cells (PBMC) from buffy coats from healthy individuals after density gradient centrifugation on lymphoprep (Fresenius Kabi Norge AS) and the use of magnetic micro bead kits (Miltenyi Biotech GmbH, Germany), according to manufacturer’s instructions. MLRs were incubated for a total of 96 hours in the presence of 5% CO_2_ and at 37°C. Eight hours before termination, 1 µCi [^3^H]-thymidine per well was added. The culture medium was complete RPMI supplemented with 50 µM 2-ME (all from Invitrogen). After being pulsed with [^3^H]-thymidine, the cells were harvested and the degree of [^3^H]-thymidine incorporation in proliferating T cells was analyzed. For ^3^H-thymidine incorporation and harvesting of cells the following material was used: ^3^H-thymidine, Tomtec harvesting machine, glass fiber filter, melt-on scintillation sheet, sample bags and 1450 Microbeta counter (Perkin Elmer, Wellesley, MA, USA). To determine whether inoculation of DC with HHV-6A can induce CD4+ T cell maturation upon co-culture, CBA targeting IL-2, IL-4, IL-6, IL-10, TNF and IFN-γ was performed on the co-culture supernatants.

To determine whether T cell proliferation in the MLRs is influenced by transmission of HHV-6A from DC to T cells, DC inoculated for 3 days with HHV-6A were co-cultured with allogenic primary CD4+ T cells in a ratio of 1∶5 in the presence of 5 ug/ml PHA-L (Roche Diagnostics GmbH, Mannheim, Germany) and 10 U/ml IL-2 (Peprotech) as previously described [21]. Infection was followed for 6 days with Q-PCR and IFA as described above. In addition to gp116/54/64 and DAPI staining in IFA, cells were also stained for CD3 using a primary polyclonal rabbit anti human CD3 MAb (Abcam, Cambridge, UK) visualized using an Alexa 488 conjugated goat anti rabbit IgG MAb (Invitrogen).

### Statistical Analysis

The data was analyzed in the GraphPad Prism v.5 software (GraphPad, La Jolla, CA, USA) using ANOVA with Bonferroni’s multiple comparison test, Student’s t-test, or two-way ANOVA (according to the data being compared) with two-tailed p-values and 95% confidence interval.

## Results

### Internalization of HHV-6A by DC is not Followed by Viral Replication

**Figure 1:**
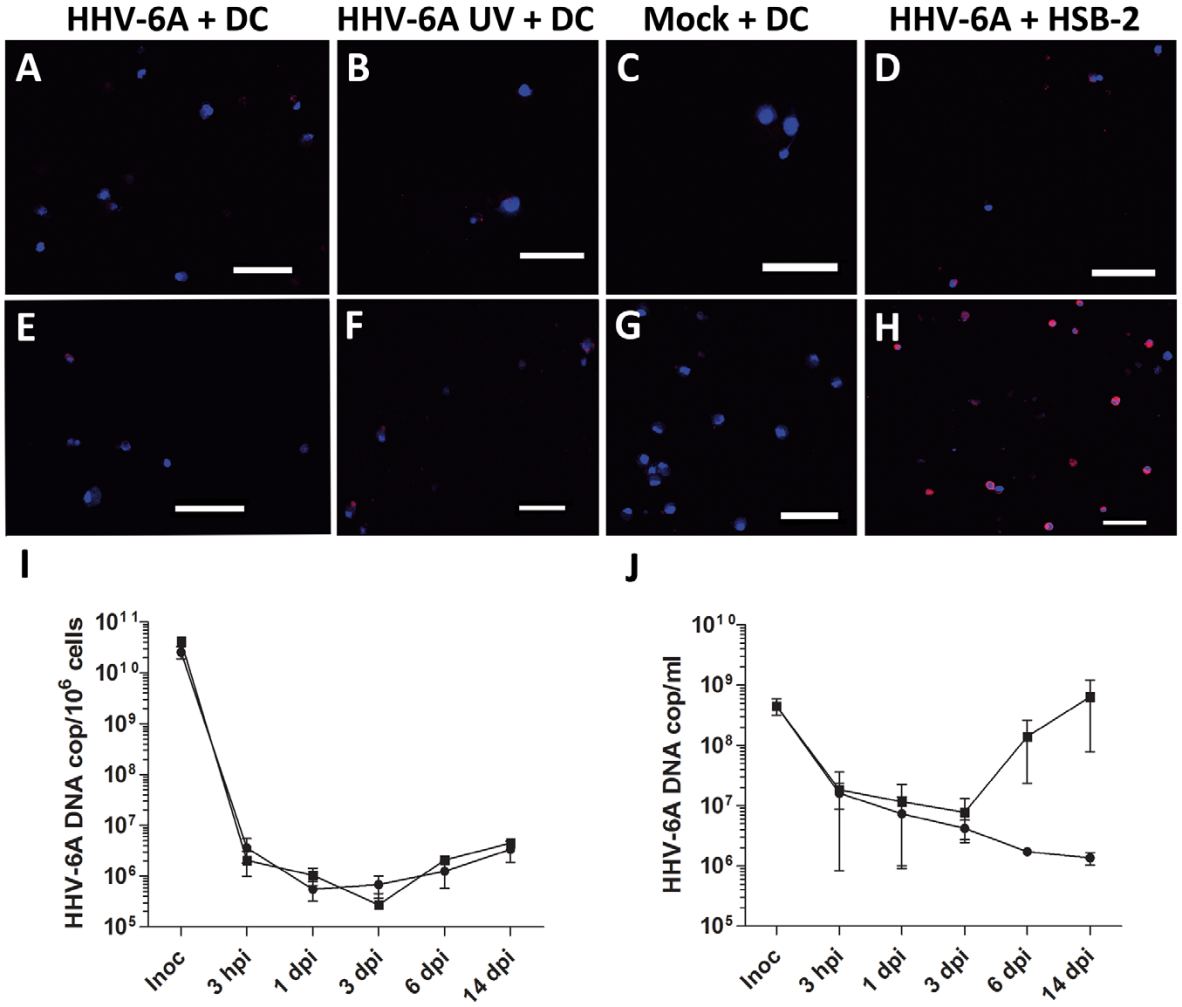
HHV-6A do not replicate in DC. DC were inoculated with 0.01 MOI of HHV-6A in untreated (A, E) or in UV-inactivated form (B–F), or with mock (C, G). As positive control HSB-2 cells were inoculated with HHV-6A at 0.01 MOI (D, H). The cells were incubated for 3 (A–D) or 6–7 days (E–H). The cells were stained with an anti-HHV-6 MAb (red) specific to the late viral protein gp116 and with DAPI (blue). Scale bars are 50 µm. Replication was also assessed by Q-PCR analysis of intracellular (I) and extracellular (J) HHV-6A DNA after inoculation of DC (filled circles) or HSB-2 cells (filled squares) with 0.01 MOI of HHV-6A, with primers and probe targeting the immediate early (IE) region of the HHV-6A genome. Data are means ± SEM for three donors.

To investigate whether HHV-6A infection of human DC leads to HHV-6A replication, DC were inoculated with HHV-6A at MOIs of 10^−1^ to 10^−4^, and mock treated cells were used as controls. DC inoculated with 0.01 MOI stained moderately positive for the late HHV-6 protein gp116/54/64 at 3–7 dpi (figures 1A and 1E), compared to mock (figures 1C and 1G). However, also DC inoculated with UV-inactivated virus, showed a similar staining pattern as observed for DC inoculated with untreated virus (figures 1B and 1F), suggesting that the viral proteins detected were not newly synthesized. In contrast, an accumulation of viral proteins was detected over time in HSB-2 cells, which supports HHV-6A replication and was used as a positive control (figures 1D and 1H). Analysis of the levels of HHV-6A DNA in cells and supernatants of DC cultures collected for up to 14 days after infection, revealed that HHV-6A persists but fails to replicate in DC (figures 1I and 1J). Thus, it is possible that human monocyte-derived DC do not allow the HHV-6A virus to replicate after the internalization.

### HHV-6A Induces Partial Activation of DC

**Figure 2:**
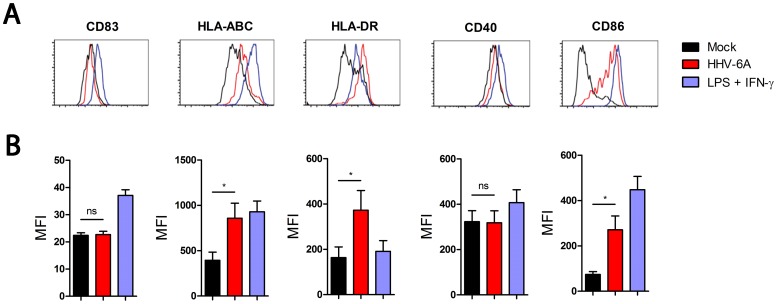
DC are partially activated by HHV-6A at 3 dpi. Representative histograms from live CD1a+ DC from one of seven donors inoculated with mock (black), HHV-6A at 0.001 MOI (red) or LPS together with IFN-γ (blue), is shown (A). Bars are means (± SEM) of mean fluorescence intensity (MFI) for seven donors. *p<0.05, ***p<0.001 using Student’s t-test (B). The flow cytometry analysis was performed using a CyAn ADP (DAKO Cytomation).

To determine whether HHV-6A inoculation of human DC induces processes of cellular activation, the surface expression of CD83, HLA-ABC, HLA-DR, CD40 and CD86 at 3 dpi on DC inoculated with HHV-6A, UV-inactivated HHV-6A, LPS and IFN-γ, or mock, were analyzed using flow cytometry. DC inoculated with HHV-6A exhibited significant up-regulation of HLA-ABC (p<0.05), HLA-DR (p<0.05) and CD86 (p<0.05) as evidenced by the higher values of mean fluorescence intensity (MFI) (figure 2B), as well as by the increased number of HHV-6A inoculated DC expressing HLA-ABC, HLA-DR and CD86 compared to the mock cells (figure 2A). In contrast, inoculation of DC with HHV-6A did not lead to significant changes in surface molecule expression of the co-stimulatory molecules CD83 and CD40 (figures 2A and 2B) Taken together these results suggest that HHV-6A partially activates DC.

### HHV-6A-induced Activation of DC Leads to IFN-α-mediated Up-regulation of HLA-ABC

**Figure 3:**
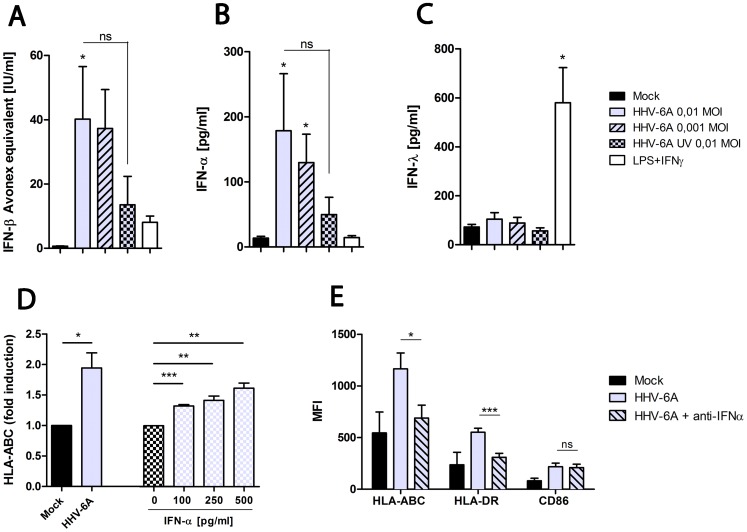
DC respond to HHV-6A (0.01 MOI) infection at 3 dpi with significantly more type I/III IFN secretion than mock, which induces HLA-ABC up-regulation. Type I/III IFN response was evaluated with an *in vitro* bioassay which measures *MxA* mRNA expression. The standard curve used is therapeutic IFNβ (Avonex®) and therefore the levels of type I IFN should be interpreted as biological effect corresponding to that of therapeutic IFNβ (A). The IFN secreted by HHV-6A inoculated DC is mainly constituted by IFN-α (B), whereas LPS and IFN-γ treated DC mainly secret IFN-λ (C). Data is shown in figures A, B and C as mean results (± SEM) for at least six donors and analyzed using one way ANOVA with Bonferroni’s multiple comparison test. DC were inoculated with 0.01 MOI of HHV-6A or mock for three hours before they were washed and cultured in complete RPMI. In parallel, DC from the same donors were cultured with 100, 250 or 500 pg IFN-α/ml. After 24 hours the cells were harvested and analyzed for HLA-ABC surface expression (D). In another experiment DC were inoculated with 0.01 MOI of HHV-6A for three hours before they were washed and cultured in complete RPMI in the presence or absence of a polyclonal anti-IFN-α antibody/ml in the culture medium. At 3 dpi the cells were harvested and analyzed for HLA-ABC, HLA-DR and CD86 surface expression (E). Data points in figures D and E are mean results (± SEM) for four donors and analyzed with Student’s t-test. *p<0.05, **p<0.01, ***p<0.001. The flow cytometry analysis was performed using a CyAn ADP (DAKO Cytomation).

Type I IFN can induce changes in DC surface phenotype, including up-regualtion of HLA and costimulatory molecules on DC [29], as well as dictate the capacity of HHV-6A to replicate in susceptible cells [30]. Therefore, we further dissected whether the interactions of HHV-6A with DC, result in production of IFN by measuring biologically active IFN. As shown in figure 3A, DC inoculated with 0.01 MOI of HHV-6A secreted significantly more IFN than mock-inoculated DC (p<0.05). Importantly, inoculation with UV inactivated HHV-6A did not induce significantly increased levels of secreted IFN compared to mock but not significantly less than with HHV-6A (figure 3A).

As *MxA* mRNA is expressed following exposure to both type I or III IFN, i.e., IFN-α and -β or -λ, respectively [27], ELISAs specific for IFN-α, -β and -λ were used to identify the nature of the IFN response induced by HHV-6A in DC. This revealed that HHV-6A inoculation of DC cultures primarily led to robust production of IFN-α (p<0.05 for inoculation with 0.01 and 0.001 MOI compared to mock) (figure 3B), while the levels of IFN-λ were unaltered as compared to mock (figure 3C) and IFN-β remained undetectable in all samples compared (data not shown). Thus, HHV-6A infection of DC predominantly induces a type I interferon response, defined as production of biologically active IFN-α.

Next, we investigated whether there is a relationship between IFN-α and the up-regulation of HLA-ABC, HLA-DR and CD86. For this purpose, exogenous IFN-α was added to DC. This revealed that HLA-ABC surface expression at 1 dpi was up-regulated (p<0.01) on DC in response to the addition of exogenous IFN-α (figure 3D), while the surface expression of HLA-DR and CD86 was not significantly affected (data not shown). To investigate whether IFN-α production by HHV-6A-inoculated DC cultures had an autocrine effect on DC maturation responses with regard to changes in surface HLA-ABC, HLA-DR and CD86, a polyclonal anti-human IFN-α antibody was added to the cultures. As shown in figure 3E, the presence of the anti-human IFN-α antibody inhibited the up-regulation at 3 dpi of both HLA-ABC (p<0.05) and HLA-DR (p<0.001) but not CD86 on DC inoculated with HHV-6A. Together, these data support a role for IFN-α in the HHV-6A-induced up-regulation of HLA-ABC on DC.

### HHV-6A Modulates Production of Inflammatory Cytokines by DC

**Figure 4:**
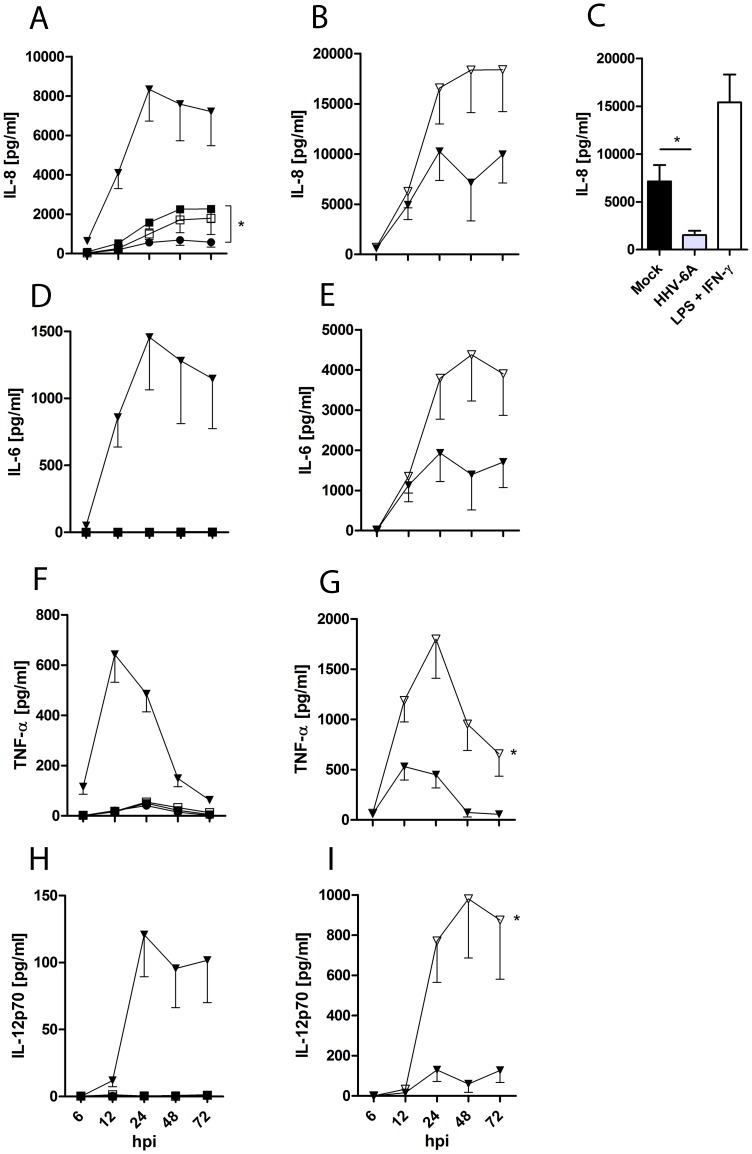
Replication capable HHV-6A reduces IL-8 production by DC but accentuates secretion of TNF and IL-12p70 by DC cultured in the presence of LPS and IFN-γ. Cytometric bead array (CBA) targeting IL-8 (A-C), IL-6 (D-E), TNF (F-G) or IL-12p70 (H-I) were performed on supernatants from DC cultures at 3 dpi. DC were inoculated with mock (open squares), 0.01 MOI of HHV-6A (filled circles) or 0.01 MOI of UV-inactivated HHV-6A (filled squares) for three hours before they were washed and cultured in complete RPMI, as positive control, DC were cultured in the presence of LPS and IFN-γ (filled triangles) (figures A, C, D, F and H). DC were inoculated with 0.01 MOI of HHV-6A (open triangles) or complete RPMI (filled triangles) for three hours before they were washed and cultured in complete RPMI containing LPS and IFN-γ (figures B, E, G and I). Data is shown as mean results (± SEM) for four donors and analyzed using two-way ANOVA for all figures except figure C were data is shown as mean results (± SEM) for at least seven donors are shown and analyzed using Student’s t-test. *p<0.05.

To further investigate whether HHV-6A can induce an inflammatory cytokine response in DC, the levels of IL-8, IL-1β, IL-6, IL-10, TNF and IL-12p70 in culture supernatants were determined using CBA. Interestingly, DC inoculated with HHV-6A secreted significantly less IL-8 than did DC inoculated with UV-inactivated HHV-6A (p<0.05) (figure 4A) or mock (p<0.05) (figure 4C). Thus, this finding suggests that active HHV-6A is capable of blocking the induction of IL-8 production by human DC. In addition, negligible levels of IL-6, TNF and IL-12p70 were produced by DC stimulated with active HHV-6A and UV-inactivated HHV-6A (figures 4D, 4F and 4H). IL-1β and IL-10 were detected at minimal levels in all cases tested (data not shown). To determine whether the blocking of IL-8 secretion by active HHV-6A in DC is sustained also in the presence of secondary stimuli, DC were inoculated for three hours with 0.01 MOI of HHV-6A, washed and cultured in the presence of LPS and IFN-γ. As shown in figure 4B, DC incubated in medium only and DC inoculated with HHV-6A were both capable of producing IL-8 in response to LPS and IFN-γ, thus, indicating that HHV-6A-mediated suppression of IL-8 was only transient. Similarly, DC inoculated with HHV-6A also secreted IL-6 in response to LPS and IFN-γ (figure 4E). In addition, DC pre-exposed to HHV-6A secreted significantly more TNF (p<0.05) and IL-12p70 (p<0.05) compared to DC incubated in medium only, in response to LPS and IFN-γ (figures 4G and 4I).

To confirm that cytokines or chemokines, which might influence phenotypic and functional properties of DC, are present only at minimal levels in the virus supernatants used for infection, CBA using two different kits was performed targeting a total of nine important cytokines in inflammation; IL-8, IL-1β, IL-6, TNF, IL-10, IL-12p70, IL-2, IL-4 and IFN-γ. The levels of all cytokines measured were similarly low or undetectable as in the supernatant of uninfected HSB-2 cells, used as mock controls in all experiments in this study. In addition, we confirmed that the virus supernatants contained only minimal levels of type I IFN and that the A549 cells, used as indicator cells, did not produce IFN *per se* in response to incubation with 100 µl of the HHV-6A supernatant. The effect in *MxA* mRNA expression in A549 cells exposed to HHV-6A infected HSB-2 supernatants was similarly low as in the cells exposed to mock-infected HSB-2 supernatants, indicating that A549 cell responses to HHV-6A are negligible (data not shown).

### HHV-6A Reduces the DC’s Allostimulatory Capacity of CD4+ or CD8+ T Cells without Viral Transmission and Promotes IL-4 Secretion

To assess whether HHV-6A can affect DC stimulation of T cells, DC inoculated with 0.01 MOI of HHV-6A or 0.01 MOI of UV inactivated HHV-6A, LPS and IFN-γ or mock were co-cultured with allogenic CD4+ or CD8+ T cells. As shown in figures 5C, HHV-6A exposed DC exhibited a reduced capacity to stimulate CD4+ T cells compared to mock and this ability was further reduced when the virus had been UV-inactivated prior to inoculation. For CD8+ T cells this effect was seen for UV-inactivated HHV-6A only (figure 5D). To investigate whether HHV-6A can induce T cell differentiation, CBA was performed on supernatants from DC and CD4+ T cell co-cultures. This revealed that IL-4 secretion was increased in co-cultures with DC that had been pre-exposed to HHV-6A compared to mock (p<0.05) (figure 5E). No differences were seen for IL-2, IL-6, IL-10, TNF or IFN-γ.

**Figure:**
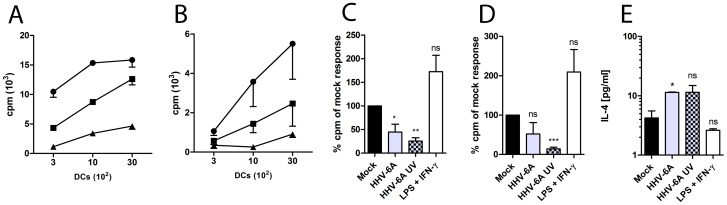
HHV-6A suppresses the capacity of DC to stimulate allogenic CD4+ T cell proliferation seen by mixed lymphocyte reactions. Incorporation of [^3^H]-thymidine into 10^5^ allogenic CD3+ CD4+ (A and C) or CD3+ CD8+ (B and D) T cells from responders was determined after four days of co-culture with DC from stimulators exposed to LPS and IFN-γ (filled circles), mock (filled squares) or 0.01 MOI of HHV-6A (filled triangles) for three days (A and B). DC from stimulators were exposed to mock, 0.01 MOI of HHV-6A, 0.01 MOI of UV-inactivated HHV-6A or LPS and IFN-γ (C and D) and 10^3^ live DC were added to each co-culture. Supernatants from co-cultures of CD3+ CD4+ T cells and DC from stimulators were harvested after four days and analyzed using CBA targeting IL-4 (E). Figures A and B show representative results of four DC donors and data points are mean results (± SEM) of counts per minutes (cpm) for triplicate wells. Figures C and D show cpm results of at least four DC donors expressed as percentage in cpm responses compared to mock stimulated DC. Figure E shows mean results (± SEM) for three donors. *p<0.05, **p<0.01 and ***p<0.01 using Student’s t-test.

To assess whether the suppression of CD4+ T cell proliferation was caused by virus transmission to the T cells from the DC, the amount of viral DNA and protein in co-cultures of HHV-6A inoculated DC and uninfected allogenic CD4+ T cells were measured by Q-PCR and IFA, respectively, for up to 6 days. This revealed that the viral DNA load decreased over time and that no viral proteins were detected at 6 dpi suggesting that HHV-6A is not transmitted from DC to the T cells (figure S1).

## Discussion

DC play an important role in initiating specific immune responses against pathogens by capturing and presenting antigens to naïve T cells. However, DC can also be targets for viral infections, and viral immune-escape mechanisms might hamper the ability of DC to activate T cells. Our results suggest that viral replication of HHV-6A is not supported by DC. However, DC becomes partially activated by HHV-6A, as evident by up-regulation of CD86 and HLA-DR, and IFN-α dependent up-regulation of HLA-ABC. In the presence of LPS and IFN-γ, HHV-6A augments the secretion of TNF and IL-12p70 by DC. Interestingly, even though DC become partially activated upon exposure to HHV-6A, they exhibit both reduced secretion of IL-8 and reduced capacity to stimulate allogenic CD4+ T cells.

Regarding the replication capacity of HHV-6A in DC [17], [22], conflicting data have been reported. In the study by Hirata *et al.* [17] it was demonstrated that DC support viral replication, as indentified by an increase in viral protein load over time after inoculation with the viral strain U1102. In contrast, the report by Smith *et al.* [22] suggests that DC do not support viral replication, as no increase in intracellular viral DNA over time after inoculation of the viral strain GS could be identified. To assess HHV-6A replication, we used both these methods, in combination with Q-PCR to detect levels of extracellular DNA after inoculation with the GS strain. Despite this systematic approach we did not see any increase in neither viral protein nor DNA (figure 1) and thus our data support the previous report by Smith *et al.* on the GS strain. The viral supernatants used by us and Smith *et al.* where indeed infectious at the time of inoculation as evident by their capacity to replicate in susceptible cells (figures 1H and 1J), supporting that the lack of viral replication is not due to degraded viral batches or suboptimal infection procedures. Instead, the varying results might be due to differences between the viral strains GS and U1102 that may affect the replication capacity in DC.

Flow cytometric analysis revealed an up-regulation of the cell surface expression of HLA–ABC, HLA-DR and CD86 on DC inoculated with HHV-6A compared to mock (figure 2). Furthermore, our data show that exogenously added IFN-α can mediate up-regulation of HLA-ABC (figure 3D) as previously reported [29]. In addition, our study suggests that IFN-α is produced by HHV-6A inoculated DC (figure 3B) and is also an important mediator of HHV-6A induced up-regulation of HLA-ABC, since the HLA-ABC expression was reduced when an anti-IFN-α antibody was added in the culture medium of the HHV-6A inoculated DC (figure 3E). Previous studies reported contradictory results on the surface expression of HLA class I on immature DC upon HHV-6A exposure. Whereas Hirata *et al.* [17] reported HLA class I down-regulation in combination with viral replication with HHV-6A U1102 in DC compared to mock, Smith *et el.* [22] saw an unaltered HLA class I expression and no viral replication with HHV-6A GS, suggesting that this down-regulation might be replication dependent, strain dependent, or both.

Type I IFN provides a number of potent antiviral effects such as induction of antibody production enhancement by CD4+ T cells and their differentiation into IFN-γ secreting Th1 cells, and the cross-priming of antigen specific CD8+ T cells [29]. HHV-6A inoculated DC responded with substantial IFN secretion compared to mock. UV-inactivated virus also induced IFN-responses, but to lower levels than DC infected with replication competent virus. This suggests that UV-treatment might destroy important viral toll like receptor (TLR) ligands or that untreated virus do replicates to low levels, under the detection limit of IFA or Q-PCR, resulting in elevated numbers of ligands accessible for TLRs in the DC. IFN-α was the major form of IFN produced by HHV-6A inoculated DC. In contrast, LPS and IFN-γ treated DC primarily produced IFN-λ (figures 3B and 3C), suggesting that HHV-6A activates other cellular signaling pathways than LPS/IFN-γ.

In the present study, HHV-6A reduced the IL-8 secretion by DC compared to UV-inactivated HHV-6A (figure 4A) or mock (figure 4C). Again, this suggests that HHV-6A might replicate at low levels and that the reduced secretion of IL-8 is dependent on replication capable virus. Assuming HHV-6A has an ability to reduce IL-8 production *in vivo* this might impair the attraction of neutrophils to the infection site, since IL-8 induces the trafficking of neutrophils across vascular walls [31]. Given that neutrophils are important cells of the immediate innate immune response [32], IL-8 down-regulation could constitute a potential immune evasion strategy of the virus. In the presence of LPS and IFN-γ however, the secretion of IL-8 by DC was slightly elevated by HHV-6A, although not significantly, suggesting that HHV-6A can reduce the IL-8 production but that this effect is overridden when other stimuli, such as LPS and IFN-γ [33], are present.

The effect of HHV-6A and HHV-6B on IL-12p70 secretion by DC is controversial [20], [22]. Our data show that HHV-6A exposure significantly increases TNF and IL-12p70 production by LPS and IFN-γ matured DC (figures 4G and 4I). HHV-6B has been shown to increase the protein expression of an LPS receptor, TLR4, in DC [34]. If this is true also for HHV-6A then an increased expression of TLR4 might enhance LPS signaling and TNF and IL-12p70 production.

Despite the virus-induced activation of DC seen in the present study, a suppressed capacity of DC to stimulate allogenic CD4+ T cell proliferation were observed upon inoculation with HHV-6A compared to mock. Interestingly, this suppressive effect was increased when the virus had been UV inactivated prior to inoculation, thus, supporting the notion that this effect was independent of productive viral replication (figure 5). Transfection of Jurkat cells with a plasmid containing the HHV-6A U24 gene (GS strain) was shown to down-regulate the T cell receptor (TCR) and CD69 expression [35]. Furthermore, non-productive infection of HHV-6B in DC could be transmitted to allogenic primary CD4+ T cells upon co-culture and converted to productive replication [21]. However, no virus transmission or replication in allogenic CD4+ T cells co-cultured with HHV-6A inoculated DC was seen in our study (figure S1), suggesting that the reduced T cell proliferation was due to direct effects on the DC rather than a viral replication effect on the T cells. Possibly, the cytokine profile secreted by the HHV-6A exposed DC, may not have been optimal to induce robust T cell proliferation but rather induce T cell differentiation. Indeed increased secretion of the T helper type 2 (Th2) cell related cytokine IL-4 [36] was seen in co-cultures of allogenic T cells with DC that had been pre-exposed to HHV-6A (figure 5E).

Taken together our results demonstrate that HHV-6A cannot replicate productively in DC. However, DC exposed to HHV-6A showed surface up-regulation of HLA-ABC mediated by autocrine IFN-α signaling, as well as increased surface expression of HLA-DR and CD86, but reduced secretion of IL-8. Furthermore, HHV-6A augmented the secretion of TNF and IL-12p70 by DC cultured in the presence of LPS and IFN-γ. Despite the activation of DC, HHV-6A or UV-inactivated HHV-6A induced a decreased capacity of DC to stimulate allogenic CD4+ T cells. The ability to suppress DC functions important for activation of adaptive immune responses might be one successful strategy by which HHV-6A avoids the induction of appropriate host defense mechanisms and thus facilitating persistent infection.

## Supporting Information

Figure S1 **HHV-6A is not transmitted from DC to T cells.** DC exposed to 0.01 MOI of HHV-6A or mock were co-cultured with allogenic CD4+ T cells at a ratio of 1∶5. Infection was followed with Q-PCR (A) and IFA for mock (B) or HHV-6A (C) exposed DC. Data shown for the Q-PCR experiments is mean results (± SEM) of four donors and for the IFA experiments representative pictures for one of four donors are shown. The cells were stained with an anti-HHV-6 MAb (red) specific to the late viral protein gp116/54/64, with DAPI (blue) and with an anti-CD3 MAb (green). Scale bars are 10 µm.
(TIF)
