# Supplementary figures and images for: Human Herpesvirus 6A Partially Suppresses Functional Properties of DC without Viral Replication
Source: PLoS One. 2013 Mar 5;8(3):e58122. doi: 10.1371/journal.pone.0058122 (PMC3590851; doi:10.1371/journal.pone.0058122)

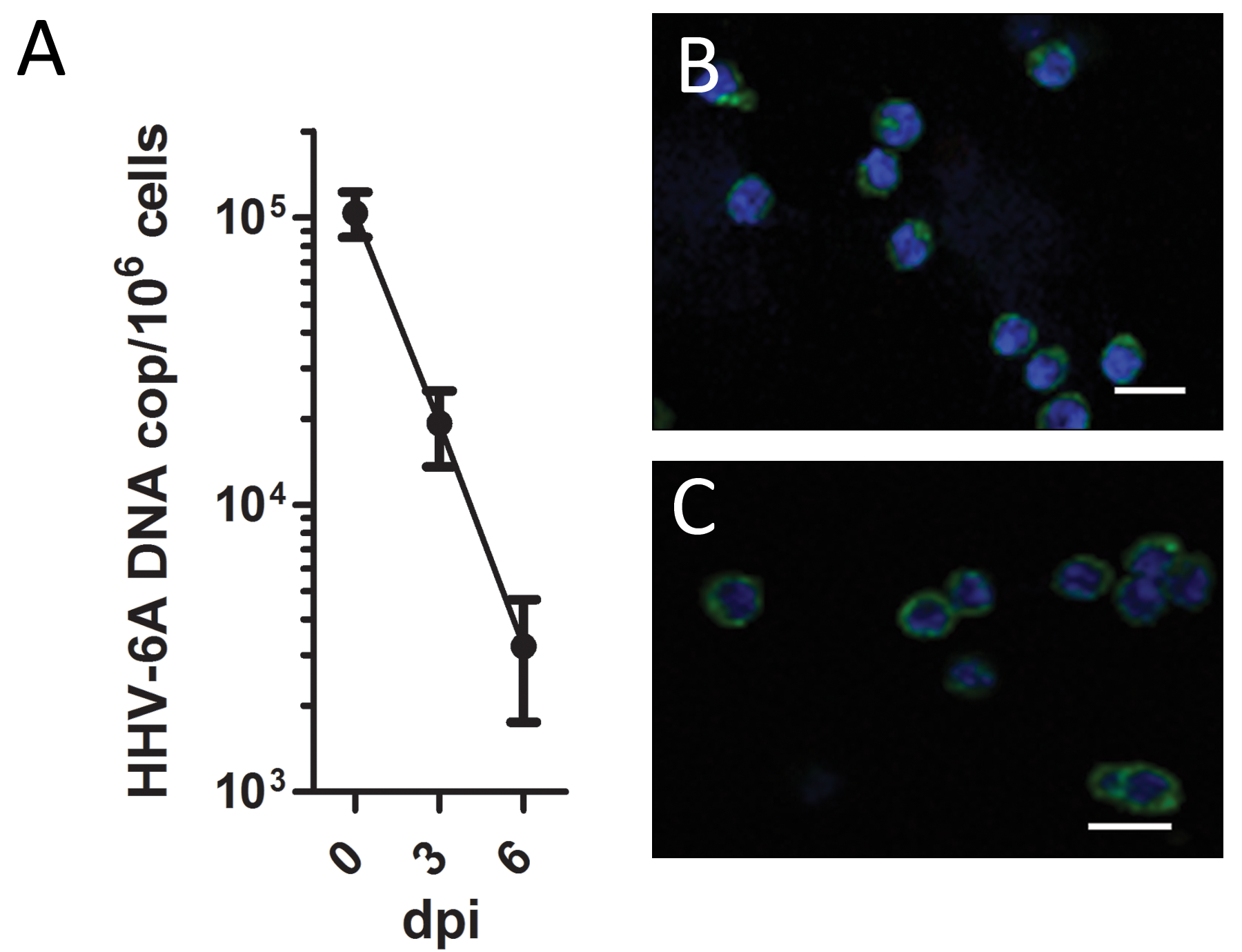

Supplement: Figure S1 — HHV-6A is not transmitted from DC to T cells. DC exposed to 0.01 MOI of HHV-6A or mock were co-cultured with allogenic CD4+ T cells at a ratio of 1∶5. Infection was followed with Q-PCR (A) and IFA for mock (B) or HHV-6A (C) exposed DC. Data shown for the Q-PCR experiments is mean results (± SEM) of four donors and for the IFA experiments representative pictures for one of four donors are shown. The cells were stained with an anti-HHV-6 MAb (red) specific to the late viral protein gp116/54/64, with DAPI (blue) and with an anti-CD3 MAb (green). Scale bars are 10 µm. (TIF) [file pone.0058122.s001.tif]
